# The awakening effect of hyperbaric oxygen therapy combined with systematic auditory stimulation in comatose patients with craniocerebral injury and its influence on serum biomarkers

**DOI:** 10.3389/fneur.2026.1775204

**Published:** 2026-05-21

**Authors:** Jie Li Rong, Yi Xiang Guan, Chun Yan Ge, Xiao Jiang Liu, Gui Lan Lu, Su Ya Cao, Cheng Guan, Rong Jin

**Affiliations:** Department of Neurosurgery, Hai'an People's Hospital, Nantong, China

**Keywords:** auditory stimulation, coma, consciousness recovery, Glasgow Outcome Scale, hyperbaric oxygen therapy, severe traumatic brain injury

## Abstract

**Background:**

Severe traumatic brain injury (sTBI) often results in prolonged coma and significant long-term disability. Evidence-based, widely accessible approaches to accelerate recovery of consciousness remain limited. This study evaluated whether adding a combined regimen of hyperbaric oxygen therapy (HBOT) and systematic auditory stimulation (SAS) to standard neurosurgical care improves recovery of consciousness and short-term outcomes in comatose patients with sTBI.

**Methods:**

In this prospective randomized controlled trial, 89 comatose patients with sTBI [Glasgow Coma Scale (GCS) < 8] were randomized to standard neurosurgical care (control, *n* = 44) or standard care in addition to HBOT and SAS (HBOT+SAS, *n* = 45). HBOT was delivered at a pressure of 2.0 atmospheres absolute (ATA) for 60 min/session, five sessions per week for 4 weeks. SAS consisted of structured family-delivered storytelling and music sessions three times daily following a predefined schedule. The primary outcome was the change in the Full Outline of UnResponsiveness (FOUR) score from baseline to Day 28. Secondary outcomes included the GCS and Coma Recovery Scale-Revised (CRS-R) scores, the Glasgow Outcome Scale (GOS) score at 3 months, and serum biomarkers [such as S100B, neuron-specific enolase (NSE), and brain-derived neurotrophic factor (BDNF)]; care-related satisfaction was analyzed as an exploratory outcome. Secondary endpoints were interpreted using a prespecified Bonferroni-adjusted threshold (*p* < 0.01).

**Results:**

Compared to the control group, the HBOT+SAS group demonstrated greater improvement in measures of consciousness at Day 28 (FOUR: 12.1 ± 2.4 vs. 9.3 ± 2.1; GCS: 11.3 ± 2.2 vs. 9.1 ± 1.9; CRS-R: 11.8 ± 2.3 vs. 8.1 ± 2.0; all *p* < 0.001). At 3 months, the functional outcome was higher in the HBOT+SAS group (GOS: 4.3 ± 0.5 vs. 3.6 ± 0.6, *p* < 0.001). Biomarker patterns indicated a reduction in neuronal injury and an enhancement in neurotrophic activity. Specifically, levels of S100B and NSE were lower in the HBOT+SAS group (both *p* < 0.001), and BDNF was higher (*p* = 0.003) in this group. No serious adverse events related to the intervention were reported. Satisfaction outcomes favored HBOT+SAS (88.89% vs. 59.09%, *p* = 0.005) and were interpreted as exploratory due to the unblinded design of the study.

**Conclusion:**

The addition of HBOT+SAS to standard care was associated with improved recovery of consciousness and better 3-month functional outcomes in comatose patients with sTBI, supported by consistent findings from clinical scales and serum biomarkers. However, since the trial did not include HBOT-only or SAS-only comparator arms, the findings support the effectiveness of the combined regimen compared to standard care but do not determine whether HBOT and SAS have a synergistic effect.

## 1 Introduction

Severe traumatic brain injury (sTBI) remains a leading cause of death and long-term disability in young and working-age populations worldwide, imposing a disproportionate burden on families and healthcare systems (1, 2). Epidemiological evidence indicates that rapid motorization and road traffic exposure have contributed to the sustained global incidence of severe head trauma (3, 4). In China, registry-based statistics report mortality rates exceeding 20% and disability rates approaching 50% among patients with sTBI, with a substantial proportion developing persistent neurological dysfunction, leading to prolonged caregiving needs and high socioeconomic costs (5). Among the aftermaths of sTBI, disorders of consciousness—particularly prolonged coma—are strongly associated with long-term dependency, diminished quality of life, and delayed rehabilitation trajectories (6–8). The time-sensitive nature of secondary brain injury further underscores the need for early, standardized management. Experimental and clinical observations suggest that severe cerebral hypoxia can trigger irreversible damage within minutes. Furthermore, clinical signs, such as bilateral pupillary dilation, are often associated with limited opportunities for meaningful neurological recovery (9, 10). Accordingly, improving early recovery of consciousness and downstream functional outcomes in comatose patients with sTBI remains a priority shared by neurosurgery and neurorehabilitation.

Current approaches to promote recovery of consciousness in patients with sTBI are multidisciplinary and include several pharmacological therapies (such as dopaminergic agents and neurotrophic medications) (11–15), neuromodulation strategies (including transcranial direct current stimulation, vagus nerve stimulation, and deep brain stimulation) (16–18), hyperbaric oxygen therapy (HBOT) (19), and rehabilitation modalities rooted in traditional Chinese medicine (20). While each approach has shown promise in selected settings, important constraints limit scalability and consistent effectiveness. Pharmacological responses are heterogeneous and may vary with injury phenotype and comorbid conditions. Neuromodulation often requires specialized equipment and expertise, restricting availability in resource-limited regions. HBOT, although biologically plausible for mitigating hypoxia-related secondary injury, may be constrained by chamber access and the logistical challenges involved in transporting patients during the acute phase (21). Notably, the majority of the existing studies has evaluated single modalities in isolation. Although multimodal stimulation paradigms have been explored—with reports of improved Glasgow Coma Scale (GCS) scores and reduced coma duration under certain combined sensory approaches (22)—the optimal combination of parameters and the extent to which observed benefits reflect component-specific effects versus bundled care remain insufficiently defined. In particular, while auditory stimulation and music-based interventions have been associated with improvements in disorders of consciousness after sTBI (23), standardized, reproducible protocols and clearer implementation frameworks are still needed.

In parallel, bedside care for comatose patients with sTBI frequently prioritizes physiological stabilization, monitoring, and complication prevention; however, structured nursing pathways focused on consciousness recovery are not uniformly implemented (24). Research in nursing systems suggests that changes in nursing models can influence critical care outcomes for patients with sTBI, including improvements in timeliness and coordination of resuscitation and ongoing management (25, 26). Responsibility-based and proceduralized nursing models may reduce response time by clarifying role allocations and streamlining workflows, thereby helping preserve a critical early window for neuroprotection. However, the integration of such structured care with sensory stimulation to form a standardized and scalable intervention for improving consciousness recovery remains incompletely addressed. From a neurophysiological perspective, multisensory stimulation is hypothesized to activate the brainstem reticular activating system through peripheral sensory inputs, which helps counteract widespread neuronal inhibition in disorders of consciousness (27, 28). Neuroimaging studies further suggest that targeted sensory stimulation can modulate regional cerebral blood flow and support compensatory functional reorganization in surviving neural networks (29, 30). Early clinical studies that combine multidimensional multisensory arousal therapy with adjunctive stimulation has reported preliminary benefits, supporting the rationale for integrated intervention strategies (31). Nonetheless, there remains a significant gap in developing a protocol that is operationally standardized, feasible in terms of resources, and suitable for routine clinical implementation.

Against this background, the present study evaluates an integrated care regimen that combines HBOT with systematic auditory stimulation (SAS) as an adjunct to conventional neurosurgical management in comatose patients with sTBI. HBOT is incorporated as a targeted physiological intervention aimed at improving tissue oxygenation and potentially mitigating hypoxia-related secondary injury. Meanwhile, SAS is implemented as a low-cost, repeatable sensory input delivered on a fixed schedule to support arousal pathways and neuroplasticity (32, 33). Importantly, the trial compares the combined HBOT+SAS regimen against standard care; thus, the study is designed to test whether this integrated approach offers clinical benefits over conventional management, rather than isolating the independent contribution of each component or establishing any mechanistic synergy. By focusing on a pragmatic and implementable combined regimen supported by standardized clinical procedures, this study aims to identify feasible strategies for improving consciousness recovery and short-term functional outcomes after sTBI. This approach has potential public health relevance as it may help reduce disability burden and strengthen major trauma rescue capabilities, aligning with the broader goals of “Healthy China 2030.”

## 2 Methods

### 2.1 Study design and participants

This prospective, single-center randomized controlled trial was conducted in the Department of Neurosurgery, Hai’an People’s Hospital. Comatose patients with severe traumatic brain injury (sTBI) were screened for eligibility. sTBI was defined as a Glasgow Coma Scale (GCS) score <8 with coma duration >30 min after injury and documented normal cognitive function before injury. Diagnosis and injury characterization were confirmed using cranial computed tomography or magnetic resonance imaging alongside standard clinical evaluation.

The study protocol involving human participants was approved by the institutional ethics committee. Because participants were comatose and lacked decision-making capacity at enrollment, written informed consent was obtained from a legally authorized representative (surrogate decision-maker) before randomization. If a participant subsequently regained decision-making capacity, consent for continued participation was reconfirmed where applicable.

**Figure 1:**
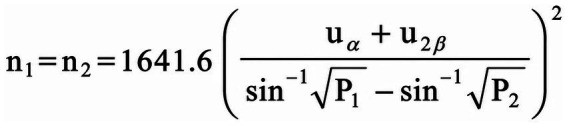
Sample size calculation formula. Sample size estimation was performed using PASS 2020 (*α* = 0.05, power = 0.90) with a 1:1 allocation ratio. The assumed proportions were P1 = 0.25 and P2 = 0.85, yielding a minimum of 40 participants per group. Accounting for ~10% attrition, the target sample size was set at 89.

Sample size estimation. Sample size was estimated using PASS 2020 with *α* = 0.05 and power (1 − *β*) = 0.90. Based on prior studies and the planned primary endpoint, the assumed proportions were P1 = 0.25 and P2 = 0.85. With a 1:1 allocation ratio, the minimum required sample size was 40 participants per group (total ≥80). Allowing for an estimated 10% attrition, the target sample size was set at 89. The formula used for the calculation is shown in Figure 1.

Eligibility criteria. Exclusion criteria included: (1) severe dysfunction of vital organs (e.g., hepatic or renal failure); (2) malignant tumors; (3) hematological or immunological disorders; (4) pre-existing neurological disorders; (5) history of blindness, deafness, psychosis, or seizures; or (6) withdrawal of surrogate consent at any time.

### 2.2 Randomization and blinding

Eligible participants were randomized in a 1:1 ratio to the control group (standard neurosurgical care) or the intervention group (standard care with hyperbaric oxygen therapy and systematic auditory stimulation, HBOT+SAS). A computer-generated random number sequence was used. Allocation concealment was ensured using sequentially numbered, opaque, sealed envelopes prepared by an independent staff member not involved in enrollment or outcome assessment. Envelopes were opened only after enrollment by a research coordinator.

**Figure 2:**
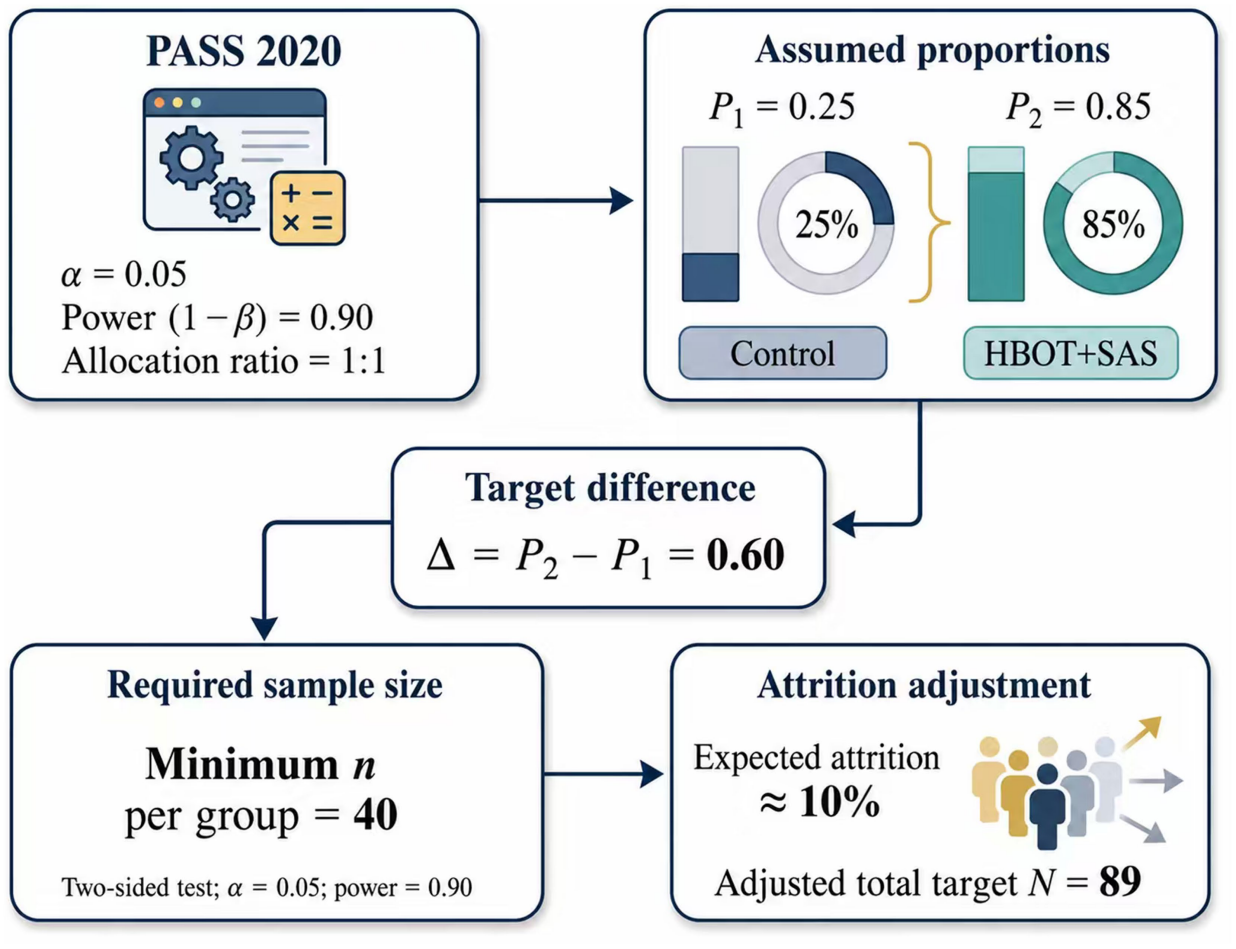
CONSORT flow diagram for participant enrollment, allocation, follow-up, and analysis. The flow diagram reports the numbers assessed for eligibility, excluded (with reasons), randomized, allocated to each group, received the allocated intervention, lost to follow-up/withdrawn (with reasons), and included in the intention-to-treat (ITT) analysis. The per-protocol (PP) set (defined *a priori* as SAS adherence ≥90%) is indicated for sensitivity analyses.

Given the nature of the interventions, blinding of treating clinicians and family caregivers was not feasible. To minimize assessment bias, outcome evaluators (neurologists/assessors) remained blinded to group assignment throughout the study period. Participant enrollment, allocation, follow-up, and analysis sets (intention-to-treat and per-protocol) are summarized in a CONSORT flow diagram (Figure 2).

### 2.3 Outcomes

The primary outcome was the change in the Full Outline of UnResponsiveness (FOUR) score from baseline to Day 28.

Secondary outcomes included the following: GCS and Coma Recovery Scale–Revised (CRS-R) scores, neurological deficit score [National Institutes of Health Stroke Scale (NIHSS); exploratory], Glasgow Outcome Scale (GOS) score at 3-month follow-up, serum biomarkers [S100B, neuron-specific enolase (NSE), and brain-derived neurotrophic factor (BDNF)] at baseline and Day 28, and nursing satisfaction score (exploratory; Supplementary material 1).

To account for multiple comparisons across secondary outcomes, a Bonferroni-adjusted significance threshold of a *p* value of <0.01 was prespecified for secondary endpoints. The primary endpoint retained a *p* value <0.05 (two-sided).

### 2.4 Interventions

#### 2.4.1 Standard neurosurgical care (control group)

Participants in the control group received routine neurosurgical and intensive care management per institutional protocols, including physiological monitoring, airway management, nutritional support, prevention of pressure injuries, and passive limb mobilization as clinically indicated.

#### 2.4.2 Combined HBOT+SAS regimen (intervention group)

In addition to standard care, participants in the intervention group received HBOT and systematic auditory stimulation (SAS) for 4 weeks.

Hyperbaric oxygen therapy (HBOT). HBOT was delivered 5 days per week for 4 consecutive weeks, 60 min per session at 2.0 ATA. Standardized safety procedures were applied for each session, including pre-session otoscopic screening, continuous monitoring of oxygenation and respiratory status during exposure, and controlled compression/decompression rates according to chamber protocols.

HBOT was initiated after initial post-admission stabilization. Participants were considered eligible to start HBOT when the treating team confirmed the following: (1) hemodynamic stability without escalating vasopressor requirements; (2) stable respiratory status with adequate oxygenation/ventilation support consistent with chamber safety protocols; (3) no evidence of uncontrolled intracranial hypertension or clinical deterioration within the preceding 24 h; and (4) no contraindications to chamber exposure, including untreated pneumothorax or other conditions judged unsafe for pressure changes. HBOT sessions were postponed or discontinued if any predefined safety concerns occurred, including hemodynamic instability; inability to maintain airway/ventilation requirements, suspected barotrauma (e.g., severe ear pain, otorrhagia, suspected tympanic membrane injury), acute neurological worsening suggestive of raised intracranial pressure, oxygen-toxicity symptoms, or any other clinician-determined contraindication.

Systematic auditory stimulation (SAS). SAS consisted of two structured components delivered three times daily: (1) family-delivered calling and storytelling following a predefined script for 30 min per session; and (2) a fixed music playlist delivered for 20 min per session at 40–60 dB (measured using a decibel meter). To support reproducibility, the script specified a stable content structure (e.g., orientation cues, familiar autobiographical references, and a neutral-to-supportive tone) and avoided distressing or highly arousing material. Stimulation was delivered in the family’s usual language or dialect familiar to the patient to maximize recognizability while maintaining the standardized structure. Family caregivers documented the content and duration of each SAS session daily using a standardized log. Nursing staff verified adherence weekly through log review and bedside checks.

Adherence and analysis set. SAS adherence was calculated as the proportion of planned sessions completed during the intervention period. All randomized participants were included in the primary analysis under an intention-to-treat (ITT) principle. A per-protocol (PP) sensitivity analysis was additionally performed among participants who achieved ≥90% SAS adherence to assess robustness; participants were not excluded from the main analysis due to adherence.

### 2.5 Grading of the severity of impaired consciousness

Consciousness was assessed at baseline and during follow-up using the FOUR and the GCS. FOUR evaluates eye response, motor response, brainstem reflexes, and respiratory pattern (each 0–4; total 0–16), with higher scores indicating better consciousness. GCS assesses eye, verbal, and motor responses (total 3–15), with higher scores indicating improved consciousness.

CRS-R was administered weekly by trained assessors blinded to allocation. CRS-R includes six subscales (auditory, visual, motor, oromotor/verbal, communication, and arousal) with a total score of 0–23, providing a sensitive measure of neurobehavioral function and recovery of consciousness.

### 2.6 Nursing satisfaction

Nursing satisfaction was assessed using the hospital’s nursing satisfaction questionnaire (Supplementary material 1). Responses were categorized as “satisfied,” “somewhat satisfied,” or “dissatisfied.” The overall satisfaction rate was calculated as: ((number satisfied + number somewhat satisfied)/total respondents) × 100%. Given the subjective nature of this outcome and the unblinded intervention context (including family participation in SAS), nursing satisfaction was treated as an exploratory endpoint.

### 2.7 Neurological deficit score (NIHSS)

NIHSS is a 12-item scale (total score 0–42) originally developed for acute stroke to quantify neurological deficits; higher scores indicate more severe impairment. In this study, NIHSS was analyzed as an exploratory indicator of neurological deficit severity. Because several items require patient cooperation, results were interpreted cautiously in comatose patients with sTBI. Scoring criteria are provided in Supplementary material 1.

### 2.8 Glasgow Outcome Scale (GOS)

Functional outcome at 3 months was assessed using the Glasgow Outcome Scale (GOS): 5 (good recovery), 4 (moderate disability), 3 (severe disability), 2 (vegetative state), and 1 (death). Higher scores indicate a better prognosis.

### 2.9 Enzyme-linked immunosorbent assay (ELISA)

Venous blood (5 mL) was collected at baseline and Day 28 into serum-separating tubes, allowed to clot for 30 min at room temperature, and centrifuged at 3,000 rpm for 15 min at 4 °C. Serum was aliquoted and stored at −80 °C until analysis.

Commercial ELISA kits were used to quantify S100B (Mlbio, Shanghai, China, #ml106520), NSE (Mlbio, Shanghai, China, #ml060406), and BDNF (Mlbio, Shanghai, China, #ml105982) according to the manufacturer’s instructions. Concentrations were calculated from standard curves.

### 2.10 Statistical analysis

Analyses were conducted using the Statistical Package for Social Sciences (SPSS version 26.0; IBM Corp., NY, USA). Continuous variables were summarized as mean ± standard deviation [or median (IQR) where appropriate] and categorical variables as *n* (%). Between-group comparisons used independent-samples *t*-tests for normally distributed continuous variables and Mann–Whitney *U* tests for non-normally distributed or ordinal variables.

Longitudinal outcomes were analyzed using linear mixed-effects models with fixed effects for group, time, and the group × time interaction, and an appropriate covariance structure to account for repeated measures. Effect sizes (e.g., Cohen’s d for continuous outcomes; rank-biserial correlation for non-parametric comparisons) with 95% confidence intervals were reported where applicable.

The primary endpoint was tested at a *p* value of <0.05 (two-sided). Secondary endpoints were interpreted using the prespecified Bonferroni-adjusted threshold of a p value of <0.01. Sensitivity analyses included a per-protocol analysis restricted to participants with SAS adherence ≥90% to evaluate robustness relative to the ITT results.

## 3 Results

### 3.1 Participant flow and baseline characteristics

During the study period, 126 patients with suspected severe traumatic brain injury (sTBI) and coma were assessed for eligibility. Thirty-seven patients were excluded before randomization: 18 did not meet inclusion criteria (e.g., GCS ≥ 8 at screening or coma duration ≤30 min), 9 had severe organ dysfunction or major comorbidities (e.g., hepatic/renal failure, malignancy, hematologic/immunologic disorders), 6 had relevant pre-existing neurological/psychiatric conditions or a history of seizures, and 4 did not proceed because surrogate consent could not be obtained. Ultimately, 89 participants were enrolled and randomized to the control group (*n* = 44) or the HBOT+SAS group (*n* = 45) (Figure 2).

All randomized participants were included in the intention-to-treat (ITT) analyses. In the HBOT+SAS group, 41 participants met the prespecified SAS adherence threshold (≥90% of planned sessions) and were included in the per-protocol (PP) sensitivity analysis; the remaining 4 participants had adherence <90% but were retained in the ITT analysis. No participants were excluded from the primary analysis due to adherence. PP sensitivity results are provided in Supplementary Table S2.

Baseline characteristics are summarized in Table 1. No statistically significant between-group differences were observed at enrollment in age, sex distribution, baseline consciousness severity (GCS and FOUR), baseline NIHSS score (exploratory), injury etiology, or time from injury to enrollment (all *p* > 0.05), supporting baseline comparability.

**Table 1:** Baseline characteristics of the study participants.

Characteristic	Control (*n* = 44)	HBOT+SAS (*n* = 45)	*p*-value
Age (years), mean ± SD	45.2 ± 11.3	43.8 ± 12.1	0.561
Male, *n* (%)	32 (72.7)	34 (75.6)	0.756
GCS, mean ± SD	6.3 ± 1.6	6.4 ± 1.7	0.770
FOUR score, mean ± SD	6.5 ± 1.7	6.8 ± 1.9	0.550
NIHSS score, mean ± SD (exploratory)	27.8 ± 5.0	27.5 ± 5.2	0.650
Etiology of injury, *n* (%)			0.607
Traffic accident	25 (56.8)	28 (62.2)	
Fall	12 (27.3)	11 (24.4)	
Blow/strike	7 (15.9)	6 (13.3)	
Time from injury to enrollment (days), mean ± SD	1.9 ± 0.7	2.0 ± 0.8	0.520

FOUR, Full Outline of UnResponsiveness; NIHSS, National Institutes of Health Stroke Scale. *p*-values were calculated using independent-samples *t*-tests for continuous variables and chi-square tests for categorical variables.

### 3.2 HBOT+SAS improves recovery of consciousness

Linear mixed-effects models demonstrated significant group × time interaction effects for all consciousness measures (Table 2). FOUR scores increased from 6.8 ± 1.9 to 12.1 ± 2.4 in the HBOT+SAS group, compared with 6.5 ± 1.7 to 9.3 ± 2.1 in the control group [*F*(1, 87) = 32.15, and *p* < 0.001; Cohen’s *d* = 1.25]. GCS increased from 6.4 ± 1.7 to 11.3 ± 2.2 in HBOT+SAS versus 6.3 ± 1.6 to 9.1 ± 1.9 in control [*F*(1, 87) = 25.67, *p* < 0.001; Cohen’s *d* = 1.08]. CRS-R also improved more in HBOT+SAS (5.4 ± 1.6 to 11.8 ± 2.3) than in the control group (5.2 ± 1.5 to 8.1 ± 2.0) [*F*(1, 87) = 28.95, *p* < 0.001; Cohen’s *d* = 1.18]. PP sensitivity analyses showed the same direction and statistical significance, supporting robustness (Supplementary Table S2).

**Table 2:** Comparison of consciousness scores before and after intervention (Day 28; ITT).

Outcome (range)	Time point	Control (*n* = 44)	HBOT+SAS (*n* = 45)	Group × time statistic	*p*-value	Effect size (95% CI)
FOUR (0–16)	Baseline	6.5 ± 1.7	6.8 ± 1.9	*F*(1, 87) = 32.15	<0.001	1.25 (0.82–1.68)
	Day 28	9.3 ± 2.1	12.1 ± 2.4			
GCS (3–15)	Baseline	6.3 ± 1.6	6.4 ± 1.7	*F*(1, 87) = 25.67	<0.001	1.08 (0.66–1.50)
	Day 28	9.1 ± 1.9	11.3 ± 2.2			
CRS-R (0–23)	Baseline	5.2 ± 1.5	5.4 ± 1.6	*F*(1, 87) = 28.95	<0.001	1.18 (0.75–1.61)
	Day 28	8.1 ± 2.0	11.8 ± 2.3			

Values are mean ± SD. Statistics derived from linear mixed-effects models (group × time interaction). Effect sizes are reported as Cohen’s *d*.

### 3.3 Nursing satisfaction (exploratory outcome)

Nursing satisfaction distributions are presented in Table 3. Using the Mann–Whitney *U* test, nursing satisfaction distributions differed between groups (*U* = 650, *p* = 0.005), with a higher overall satisfaction rate in HBOT+SAS (88.89%) than in the control (59.09%). The effect size was moderate, with a rank-biserial correlation (*r* = 0.33, 95% CI, 0.10–0.53). Given that this measure is subjective and collected in an unblinded context with family involvement in SAS delivery, nursing satisfaction was prespecified as an exploratory endpoint and interpreted cautiously.

**Table 3:** Comparison of nursing satisfaction (exploratory; ITT).

Group	Satisfied (*n*)	Somewhat satisfied (*n*)	Dissatisfied (*n*)	Satisfaction rate (%)	Statistic	*p*-value	Effect size (95% CI)
Control (*n* = 44)	17	9	18	59.09	*U* = 650	0.005	0.33 (0.10–0.53)
HBOT+SAS (*n* = 45)	27	13	5	88.89			

*p*-values derived from the Mann–Whitney *U* test. Effect size is the rank-biserial correlation (*r*). Questionnaire details are provided in Supplementary material 1.

### 3.4 Neurological function (NIHSS; exploratory)

NIHSS scores are presented in Table 4. Linear mixed-effects models indicated a significant group × time interaction [*F*(1, 87) = 21.04, *p* < 0.001]. NIHSS decreased from 27.5 ± 5.2 to 14.2 ± 4.1 in the HBOT+SAS group compared with 27.8 ± 5.0 and 20.5 ± 4.3 in the control group (Cohen’s *d* = −1.12, 95% CI, −1.54 to −0.70). NIHSS was prespecified as an exploratory measure and is interpreted cautiously in comatose patients with sTBI due to cooperation-dependent items.

**Table 4:** Comparison of NIHSS scores before and after intervention (exploratory; Day 28; ITT).

Outcome (range)	Time point	Control (*n* = 44)	HBOT+SAS (*n* = 45)	Group × time statistic	*p*-value	Effect size (95% CI)
NIHSS (0–42)	Baseline	27.8 ± 5.0	27.5 ± 5.2	*F*(1, 87) = 21.04	<0.001	−1.12 (−1.54 to −0.70)
	Day 28	20.5 ± 4.3	14.2 ± 4.1			

Values are mean ± SD. Statistics derived from linear mixed-effects models (group × time interaction). Effect sizes are reported as Cohen’s *d*.

### 3.5 Functional outcome at 3 months (GOS)

At 3-month follow-up, GOS scores were higher in the HBOT+SAS group than in the control group (Mann–Whitney *U* = 530, *p* < 0.001; Table 5). The mean GOS score was 4.3 ± 0.5 in HBOT+SAS versus 3.6 ± 0.6 in the control group, with a large effect size (rank-biserial correlation *r* = 0.55, 95% CI, 0.36–0.70).

**Table 5:** Comparison of GOS scores at 3-month follow-up (ITT).

Outcome	Time point	Control (*n* = 44)	HBOT+SAS (*n* = 45)	Statistic	*p*-value	Effect size (95% CI)
GOS	3-month follow-up	3.6 ± 0.6	4.3 ± 0.5	*U* = 530	<0.001	0.55 (0.36–0.70)

*p*-values derived from the Mann–Whitney *U* test. Effect sizes are reported as rank-biserial correlation (*r*).

### 3.6 Serum biomarkers

Serum biomarker results are summarized in Table 6. Linear mixed-effects models showed significant group × time interactions for S100B [*F*(1, 87) = 26.78, *p* < 0.001], NSE [*F*(1, 87) = 21.91, *p* < 0.001], and BDNF [*F*(1, 87) = 9.62, *p* = 0.003]. Under the prespecified Bonferroni-adjusted threshold for secondary endpoints (*p* < 0.01), all three biomarker results remained statistically significant. From baseline to Day 28, S100B and NSE decreased more in the HBOT+SAS group, while BDNF increased more in HBOT+SAS compared with the control group.

**Table 6:** Comparison of serum biomarker concentrations before and after intervention (Day 28; ITT).

Biomarker	Time point	Control (*n* = 44)	HBOT+SAS (*n* = 45)	Group × time statistic	*p*-value	Effect size (95% CI)
S100B (ng/mL)	Baseline	0.87 ± 0.16	0.85 ± 0.15	*F*(1, 87) = 26.78	<0.001	−1.10 (−1.52 to −0.68)
	Day 28	0.65 ± 0.12	0.45 ± 0.10			
NSE (ng/mL)	Baseline	25.6 ± 3.3	25.4 ± 3.2	*F*(1, 87) = 21.91	<0.001	−1.00 (−1.42 to −0.58)
	Day 28	20.3 ± 3.0	15.2 ± 2.5			
BDNF (pg/mL)	Baseline	122 ± 21	120 ± 20	*F*(1, 87) = 9.62	0.003	0.66 (0.25–1.07)
	Day 28	130 ± 22	150 ± 25			

Values are mean ± SD. Statistics derived from linear mixed-effects models (group × time interaction). Effect sizes are reported as Cohen’s *d*.

### 3.7 Safety and adverse events

No serious adverse events related to the combined regimen were observed during the study period. In the HBOT+SAS group, two patients (4.4%) experienced transient middle-ear discomfort during the compression phase of HBOT. Symptoms resolved after reducing the compression rate and applying standard equalization procedures. No cases of oxygen toxicity or tympanic membrane injury were reported.

## 4 Discussion

Severe traumatic brain injury (sTBI) leading to prolonged coma substantially delays neurofunctional rehabilitation and imposes a heavy caregiving burden on families and society. Although clinical practice often combines pharmacologic agents, hyperbaric oxygen therapy, and environmental stimulation approaches, outcomes related to recovery-of-consciousness remain unsatisfactory. In this context, we evaluated a pragmatic integrated regimen combining hyperbaric oxygen therapy (HBOT) and systematic auditory stimulation (SAS) and observed greater improvements in consciousness-related scales, medium-term functional outcomes, and serum biomarkers compared with standard care alone. Because this trial compared the combined regimen versus standard care and did not include HBOT-only or SAS-only arms, the findings should be interpreted as evidence of combined efficacy, rather than definitive proof that the two components act synergistically beyond additive effects.

First, scale-based assessments consistently indicated better recovery of consciousness in the HBOT+SAS group. Patients receiving the combined regimen showed larger gains in FOUR and GCS scores than the control group, suggesting a more pronounced improvement in responsiveness and consciousness level. These findings were corroborated by CRS-R, which is widely used in disorders of consciousness and is more sensitive to subtle neurobehavioral changes than more global scales. The significant group × time interaction in CRS-R supports the association between HBOT+SAS and enhanced recovery of conscious awareness during early post-injury care. From a biological plausibility perspective, HBOT may alleviate cerebral tissue oxygen metabolism crises and improve tissue oxygen availability in the injured brain, potentially mitigating secondary injury cascades related to hypoxia (34, 35). In parallel, structured auditory stimulation may activate auditory pathways and limbic-affective circuits and facilitate arousal-related network engagement, which together provide a reasonable rationale for the combined regimen (36, 37). These complementary pathways may help explain why the combined regimen was associated with better clinical trajectories, while the current design does not permit formal inference about mechanistic interaction.

Second, NIHSS scores decreased more in the HBOT+SAS group. However, NIHSS was originally developed and validated for stroke populations and includes cooperation-dependent items, which limit its interpretability in comatose patients with sTBI. Accordingly, NIHSS should be considered an exploratory indicator in the present study, and its results should be interpreted cautiously. Even so, the direction of change is compatible with prior evidence that HBOT can reduce cerebral edema and may support angiogenesis and tissue repair in brain injury contexts (38, 39), while repeated auditory stimulation may contribute to experience-dependent plasticity. Future studies should prioritize outcome measures that are more directly validated for severe TBI and disorders of consciousness and treat NIHSS as a methodological limitation rather than a primary indicator of neurological recovery.

Third, biomarker monitoring provided biological correlates that align with the observed clinical improvements. The reductions in S100B and NSE in the HBOT+SAS group suggest attenuation of injury-associated signaling and may reflect changes in blood–brain barrier disruption and neuronal/glial injury processes. S100B has also been discussed as having concentration-dependent biological effects, with potential neurotrophic roles at low concentrations (40). During hyperbaric oxygen therapy, improved systemic oxygenation may support oxidative phosphorylation and energy metabolism and may modulate pathways involved in inflammation and oxidative stress (e.g., NF-κB, p38 MAPK), potentially reducing downstream neurotoxicity associated with secondary injury cascades (41, 42). Meanwhile, the increase in BDNF observed in the HBOT+SAS group is consistent with the role of BDNF in synaptic plasticity, neurogenesis, and maintenance of neuronal viability, processes that are relevant to recovery after severe brain injury (43–45). While these biomarker patterns strengthen biological plausibility, they should be interpreted as supportive evidence rather than definitive proof of a specific causal mechanism or synergy.

Fourth, the early gains in consciousness and neurological trajectories were accompanied by improved 3-month functional outcomes, as reflected by higher GOS scores in the HBOT+SAS group. Earlier recovery of arousal and responsiveness may reduce the risk of coma-related complications, including muscle atrophy and joint contractures, and may expand opportunities for timely rehabilitation engagement (46). From a care-delivery perspective, nursing satisfaction was higher in the intervention group, suggesting acceptability of the combined regimen among families and caregivers. However, nursing satisfaction is subjective and vulnerable to bias in unblinded settings—particularly when family members participate in delivering auditory stimulation—so it should be interpreted as an indicator of feasibility and perceived care experience rather than a primary efficacy endpoint.

Several limitations should be acknowledged. First, this was a single-center trial, which limits generalizability across healthcare systems and populations. Second, the absence of HBOT-only and SAS-only comparator arms prevents attribution of observed effects to either component alone and does not allow formal testing of additive versus synergistic effects. Third, the follow-up duration was limited to 3 months, which may be insufficient to characterize longer-term neurological recovery trajectories and functional independence after sTBI. Fourth, although outcome assessors were blinded to group allocation, the interventions themselves could not be blinded, and subjective outcomes (especially nursing satisfaction) remain susceptible to expectation and performance effects. Finally, the post-randomization adherence threshold was handled as a PP sensitivity analysis rather than an exclusion criterion in the primary ITT analyses, but future trials would benefit from prespecified adherence-support strategies and attention-matched comparators to reduce performance-related bias.

In future work, multicenter trials with larger sample sizes, longer follow-up (e.g., ≥12 months), and factorial or multi-arm designs including HBOT-only and SAS-only groups are needed to disentangle component-specific effects and clarify whether interaction effects exist. Further standardization of SAS content (e.g., emotional valence, language, familiarity) and reporting of cultural variability would also strengthen reproducibility and interpretability. Overall, the present findings support HBOT+SAS as a clinically feasible combined regimen associated with improved recovery of consciousness and functional outcomes compared with standard care, while emphasizing that potential mechanistic synergy remains to be tested in appropriately designed trials.

## Data Availability

The raw data supporting the conclusions of this article will be made available by the authors, without undue reservation.
