# Supplementary material for: The awakening effect of hyperbaric oxygen therapy combined with systematic auditory stimulation in comatose patients with craniocerebral injury and its influence on serum biomarkers
Source: Front Neurol. 2026 May 21;17:1775204. doi: 10.3389/fneur.2026.1775204 (PMC13233257; doi:10.3389/fneur.2026.1775204)
Supplement: Supplementary file 1 [file Table_1.docx]

Supplementary Material 1

Supplementary Table S1A. Nursing Satisfaction Questionnaire (Institutional 3-point Survey)

Purpose: Exploratory assessment of caregivers’ satisfaction with nursing care during hospitalization.

Respondent: Primary caregiver / legally authorized representative (LAR) who was most involved in bedside communication and care coordination.

Administration timepoint: Day 28 post-randomization or at discharge (whichever occurred first).

Collector: A ward nurse not involved in blinded outcome scoring (FOUR/GCS/CRS-R) to reduce assessment bias.

Response format: Single-choice, three-category response for each item.

Response options (tick one):

☐ Satisfied  ☐ Somewhat satisfied  ☐ Dissatisfied

Item No. Domain Question Response (choose one)

1 Communication The nursing team provided clear and timely updates about the patient’s condition and care plan. ☐ Satisfied ☐ Somewhat satisfied ☐ Dissatisfied

2 Responsiveness Nurses responded promptly when assistance or information was needed. ☐ Satisfied ☐ Somewhat satisfied ☐ Dissatisfied

3 Professional competence Nursing procedures were performed in a professional and reassuring manner. ☐ Satisfied ☐ Somewhat satisfied ☐ Dissatisfied

4 Safety and monitoring I felt confident in the monitoring and safety management during hospitalization. ☐ Satisfied ☐ Somewhat satisfied ☐ Dissatisfied

5 Overall satisfaction Overall, I am satisfied with the nursing care received. ☐ Satisfied ☐ Somewhat satisfied ☐ Dissatisfied

Derived indicator and calculation:

Overall satisfaction rate (%) = (No. “Satisfied” + No. “Somewhat satisfied”) / Total respondents × 100.00%.

Interpretation note (for reviewers):

This institutional survey is subjective and collected in an unblinded context; therefore, it is treated as an exploratory outcome and interpreted cautiously.

Supplementary Table S1B. NIHSS Scoring Framework and Rater Standardization (Exploratory)

Outcome role in this trial: Exploratory neurological deficit measure (secondary/exploratory), interpreted cautiously in comatose sTBI patients because several items require cooperation.

Rater: Trained neurologist/assessor blinded to group allocation.

Assessment schedule: Baseline and Day 28 (align with main manuscript).

Training/standardization: Raters completed standardized NIHSS training and followed standard scoring rules. If an item was not testable due to the level of consciousness or clinical constraints, it was scored using NIHSS standard “untestable” guidance, and the reason was documented.

NIHSS item structure (overview): NIHSS includes 12 domains with a total score range of 0–42 (higher scores indicate more severe deficit). The domains include:

1. Level of consciousness (LOC)

2. LOC questions

3. LOC commands

4. Best gaze

5. Visual fields

6. Facial palsy

7. Motor arm

8. Motor leg

9. Limb ataxia

10. Sensory

11. Best language

12. Dysarthria

13. Extinction and inattention (note: some NIHSS versions enumerate as 11/12/13; scoring followed the version used in training)

Total score interpretation:

0 = no deficit; higher scores = more severe neurological impairment.

Copyright and form availability note:

The official NIHSS scoring sheet is a standardized instrument distributed with authorized training materials and is not reproduced verbatim here. Scoring in this study followed the standard NIHSS rules used in routine clinical practice and documented in rater training resources.

Supplementary Table S1C. Systematic Auditory Stimulation (SAS) Session Log and Adherence Calculation

Purpose: To standardize documentation of SAS delivery and to support adherence calculation for per-protocol sensitivity analysis (≥90% adherence), while maintaining intention-to-treat as the primary analysis set.

S1C-1. Daily SAS Log (to be completed by caregiver; verified weekly by nursing staff)

Patient Study ID: ________  Date: ____ / ____ / ____

Caregiver/LAR initials: ________  Verifier (nurse initials): ________

A. Storytelling/Calling Session (target: 30 min per session; 3 sessions/day)

For each session, tick completion and record duration/content category.

Session Completed (Y/N) Start time End time Duration (min) Content category* Notes (optional)

Morning ☐Y ☐N ____ ____ ____ ☐ Orientation ☐ Autobiographical ☐ Neutral/supportive ____

Afternoon ☐Y ☐N ____ ____ ____ ☐ Orientation ☐ Autobiographical ☐ Neutral/supportive ____

Evening ☐Y ☐N ____ ____ ____ ☐ Orientation ☐ Autobiographical ☐ Neutral/supportive ____

*Content category definitions (for standardization):

• Orientation: name, date/time cues, location, simple factual reminders

• Autobiographical: familiar family events, routines, personally relevant memories

• Neutral/supportive: calm encouragement, neutral narration; avoid highly distressing topics

B. Music Session (target: 20 min per session; 3 sessions/day; volume 40–60 dB)

Session Completed (Y/N) Playlist ID Measured dB (start/mid/end) Duration (min) Notes (optional)

Morning ☐Y ☐N ____ ____ / ____ / ____ ____ ____

Afternoon ☐Y ☐N ____ ____ / ____ / ____ ____ ____

Evening ☐Y ☐N ____ ____ / ____ / ____ ____ ____

Weekly verification statement (nurse):

I verified the SAS log entries against bedside checks and caregiver clarification.

Nurse initials/signature: ________  Date: ____ / ____ / ____

S1C-2. Adherence calculation rule (used for PP sensitivity analysis)

• Planned SAS sessions per day: 6 total (3 storytelling + 3 music)

• Planned sessions during 4 weeks: 6 × 28 = 168 sessions (adjust if your protocol used different duration/days)

• Completed sessions: counted when recorded as “Completed = Y” with a duration ≥80% of target time (≥24 min storytelling; ≥16 min music) OR specify your operational rule if different.

• Adherence (%) = Completed sessions / Planned sessions × 100

• Per-protocol threshold: ≥90% adherence

Note: Participants are not excluded from the primary ITT analysis due to adherence; adherence is used only for PP sensitivity analysis.
