# Supplementary material for: The awakening effect of hyperbaric oxygen therapy combined with systematic auditory stimulation in comatose patients with craniocerebral injury and its influence on serum biomarkers
Source: Front Neurol. 2026 May 21;17:1775204. doi: 10.3389/fneur.2026.1775204 (PMC13233257; doi:10.3389/fneur.2026.1775204)
Supplement: Supplementary file 2 [file Table_2.docx]

Supplementary Material 2

Supplementary Table S2. Per-protocol (PP) sensitivity analyses (HBOT+SAS adherence ≥90%)

PP definition: HBOT+SAS participants with ≥90% planned SAS sessions completed (n=41).

Primary analysis: ITT (Control n=44; HBOT+SAS n=45). PP is sensitivity only.

| **Outcome** | **Time-point** | **ITT Control** $\text{n}\text{=44}$ **mean ± SD** | **ITT HBOT+SAS** $\text{n}\text{=45}$ **mean ± SD** | **PP HBOT+SAS** $\text{n}\text{=41}$ **mean ± SD** | **Statistical approach** | **Consistency statement** |
| --- | --- | --- | --- | --- | --- | --- |
| FOUR (0–16) | Baseline | 6.5 ± 1.7 | 6.8 ± 1.9 | 6.9 ± 1.8 | LMM (group×time) | Direction consistent with ITT |
|  | Day 28 | 9.3 ± 2.1 | 12.1 ± 2.4 | 12.4 ± 2.3 |  |  |
| GCS (3–15) | Baseline | 6.3 ± 1.6 | 6.4 ± 1.7 | 6.5 ± 1.6 | LMM (group×time) | Direction consistent with ITT |
|  | Day 28 | 9.1 ± 1.9 | 11.3 ± 2.2 | 11.6 ± 2.1 |  |  |
| CRS-R (0–23) | Baseline | 5.2 ± 1.5 | 5.4 ± 1.6 | 5.5 ± 1.5 | LMM (group×time) | Direction consistent with ITT |
|  | Day 28 | 8.1 ± 2.0 | 11.8 ± 2.3 | 12.1 ± 2.2 |  |  |
| NIHSS (0–42) (exploratory) | Baseline | 27.8 ± 5.0 | 27.5 ± 5.2 | 27.3 ± 5.1 | LMM (group×time) | Direction consistent with ITT (exploratory) |
|  | Day 28 | 20.5 ± 4.3 | 14.2 ± 4.1 | 13.8 ± 4.0 |  |  |
| S100B (ng/mL) | Baseline | 0.87 ± 0.16 | 0.85 ± 0.15 | 0.84 ± 0.15 | LMM (group×time) | Direction consistent with ITT |
|  | Day 28 | 0.65 ± 0.12 | 0.45 ± 0.10 | 0.44 ± 0.10 |  |  |
| NSE (ng/mL) | Baseline | 25.6 ± 3.3 | 25.4 ± 3.2 | 25.3 ± 3.1 | LMM (group×time) | Direction consistent with ITT |
|  | Day 28 | 20.3 ± 3.0 | 15.2 ± 2.5 | 15.0 ± 2.4 |  |  |
| BDNF (pg/mL) | Baseline | 122 ± 21 | 120 ± 20 | 121 ± 20 | LMM (group×time) | Direction consistent with ITT |
|  | Day 28 | 130 ± 22 | 150 ± 25 | 152 ± 24 |  |  |
| Nursing satisfaction (exploratory) | Day 28/discharge | 59.09% | 88.89% | 90.24% | Mann–Whitney U | Exploratory; unblinded |
